# Evaluating the Feasibility of Pro-Neurotensin and 25-Hydroxyvitamin D3 as Possible Indicators for Type 2 Diabetes Mellitus and Its Complications

**DOI:** 10.3390/healthcare11081088

**Published:** 2023-04-11

**Authors:** Amal A. Mohammed, Dina M. Abo El-Matty, Esraa A. Abd ElSalam, Mona A. Hussein, Wael Hafez, Sharehan A. Ibrahim, Eman A. H. Shaheen, Eman A. Awad, Marwa A. Osman, Marwa S. Abd El-Raouf, Salma M. Saed, Reham Y. El-Amir, Doaa Ghaith, Fatme Al Anouti, Alaa S. Wahba

**Affiliations:** 1 Department of Biochemistry and Molecular Biology, National Hepatology and Tropical Medicine Research Institute (NHTMRI), Cairo 11796, Egypt; 2 Department of Biochemistry and Molecular Biology, Faculty of Pharmacy, Suez Canal University, Ismailia 41522, Egypt; 3 Department of Internal Medicine, National Institute of Diabetes and Endocrinology, Cairo 11562, Egypt; 4 Department of Internal Medicine, The National Research Centre, Cairo 11511, Egypt; waeelhafez@yahoo.com; 5 Department of Internal Medicine, Faculty of Medicine, Minia University, Minya 61511, Egypt; 6 Department of Clinical and Chemical Pathology, Faculty of Medicine, Helwan University, Cairo 11795, Egypt; 7 Department of Internal Medicine, Faculty of Medicine for Girls, Al-Azhar University, Cairo 11795, Egypt; 8 Department of Neurology, Faculty of Medicine for Girls, Al-Azhar University, Cairo 11884, Egypt; 9 Department of Public Health, Faculty of Medicine, Benha University, Benha 13511, Egypt; 10 Department of Clinical and Chemical Pathology Department, Faculty of Medicine, Cairo University, Cairo 11566, Egypt; 11 Department of Public Health, Faculty of Medicine, Cairo University, Giza 12211, Egypt; 12 Department of Health Sciences, College of Natural and Health Sciences, Zayed University, Abu Dhabi 144534, United Arab Emirates

**Keywords:** 25 (OH) Vit D3, Pro-NT, marker, insulin resistance, T2DM

## Abstract

(1) Background: Type 2 diabetes mellitus (T2DM) and metabolic syndrome are associated with decreased vitamin D. In contrast, high pro-neurotensin (pro-NT) levels are linked with an increased risk of T2DM and cardiovascular disease. We aimed to determine the validity of pro-NT and 25-dihydroxy vitamin D3 levels as predictors for T2DM complications; (2) Methods: One hundred T2DM, and one hundred healthy volunteers participated in this case-control study. Their Pro-NT and 25-hydroxyvitamin D3 levels were evaluated using the ELISA technique; (3) Results: Pro-NT and 25 (OH) vitamin D3 have significant validity and accuracy in T2DM prediction, 84.5%, and 90.5%, respectively (*p* = 0.001). At a value of <29.5, 25-Hydroxy vitamin D3 showed 88% sensitivity and 93% specificity in predicting T2DM. At a value of >124 Pmol/L, Pro-NT showed 81% sensitivity and 88% specificity in predicting T2DM. At a value of 16.5, 25-Hydroxy vitamin D3 had 78.4% sensitivity and 68.3% specificity in predicting T2DM complications. At a value of >158 pmol/L, Pro-NT predicted T2DM complications with 67.6% sensitivity and 56.0% specificity; (4) Conclusions: 25 (OH) Vit D3 and Pro-NT could identify T2DM patients and predict T2DM complications. More extensive research is required to adequately validate this novel perspective with a large population study.

## 1. Introduction

Type 2 diabetes mellitus (T2DM) is a metabolic condition characterized by hyperglycemia caused by a combination of pathophysiological variables, primarily insulin resistance and insufficient insulin production [1,2]. Research indicates that obesity is a potential aspect of T2DM [3], while other evidence hypothesizes that obesity is predictable in T2DM [4]. Insulin resistance is linked to both obesity and type 2 diabetes. The inability of β-cells to properly compensate for decreased insulin sensitivity is a necessary condition for obesity and insulin resistance to be linked to type 2 diabetes. Even when the patient’s blood glucose levels are within normal range, beta-cell dysfunction can occur in those at high risk of developing the condition. Inducing insulin resistance and impairing cell function, non-esterified fatty acids (NEFAs) are a plausible offender [3]. According to International Diabetes Federation (IDF), Egypt is among the top 10 nations with a high prevalence of diabetes, with around 10.9 million Egyptians having diabetes and another 6.8 million going undiagnosed. Moreover, the IDF also reported that diabetes-related mortality reached 122,684 in Egypt in 2021. The global prevalence of DM was 10.5% by 2021 and is predicted to reach 12.2% by 2045 [5]. Despite this, investigations examining the prospective risk of DM, or its component pathological factors are still lacking. 

Neurotensin (NT) is a 13 amino acid peptide, released from the enteroendocrine N cells of the small bowel in response to fat intake [6], and is emitted from CNS [7]. NT increases intestinal fat absorption [8] and is shown centrally to regulate satiety [9]. Human and animal trials indicated the association between obesity and NT [8,10]. In vivo and in vitro, it is difficult to measure NT as it is unstable. However, a stable and measurable 117-amino acid fragment is proneurotensin (pro-NT), which is emitted along with the mature hormone in a 1:1 ratio [11]. Increased plasma levels of proneurotensin are linked to a high incidence of DM, cardiovascular disease, and mortality [12]; however, there are also reports which contradict these findings [13,14]. Coronary artery disease is associated with genetic variants of the neurotensin receptor 3 (NTSR3), and it has even been proposed that NTSR3 regulates GLUT4, a major glucose transporter in peripheral muscle and adipose tissue that controls insulin sensitivity [13]. Through mechanisms related to insulin resistance [15], Fawad et al. have demonstrated that elevated plasma neurotensin levels in dysmetabolic people can detect the prevalence and severity of NAFLD [13]. These results point to a key involvement for the proneurotensin system (neurotensin, NTSR1 and NTSR3) in the development of T2D through influencing insulin resistance and glucose regulation [12].

Vitamin D insufficiency is a frequent condition in healthy Egyptians [16]. Low levels of vitamin D are related to obesity, hypertension, hyperlipidemia, glucose intolerance, cardiovascular disease, and a high risk of metabolic syndrome [17,18,19,20]. Vitamin D has a crucial role in T2DM pathogenesis by regulating insulin receptor expression of genes and their secretion [21]. One study demonstrates a correlation between low serum levels of vitamin D and an increased risk of T2DM [22]. Moreover, several studies support the theory that sufficient vitamin D has a beneficial effect on glycemic regulation [23,24]. As serum 25-hydroxyl vitamin D (25 (OH) D3), the main circulating form of vitamin D, has a lower clearance capacity than 1, 25 (OH)2 D3, it is a good predictor for vitamin D supplementation [25,26]. Insulin secretion and insulin resistance, two crucial processes connected to type 2 diabetes, are dependent on vitamin D levels. The vitamin D receptor and 1-hydroxylase enzyme, which convert 25 (OH) D into the active hormone 1, 25-dihydroxyvitamin D, are found in beta cells. Additionally, in vitro, and in vivo studies show that vitamin D receptor knockout or vitamin D deficiency impairs glucose-induced insulin secretion, and the insulin secretory response improves after vitamin D supplementation in both animals and humans. These findings support a role for vitamin D in insulin secretion. The presence of the vitamin D receptor in skeletal muscle cells, stimulation of insulin receptor expression and insulin-induced glucose transport in vitro, and direct regulation of pathways involved in the regulation of fatty acid metabolism in skeletal muscle and adipose tissue are all evidence that vitamin D plays a role in insulin sensitivity [22].

Both neurotensin and vitamin D are linked with the development of T2DM and its complications among several ethnic groups. To our knowledge, our current study is the first study on this issue. We aim to assess the relationship between vitamin D and proneurotensin concentrations as a predictor of T2DM and its complications in a case-control study setting on type 2 diabetic Egyptian patients.

## 2. Materials and Methods

### 2.1. Study Population and Design

This case-control study included 200 participants, all of them more than 22 years old, of which 100 were T2DM patients and the rest were healthy (control group). More specifically, we studied type 2 diabetic patients (males, 63; females, 37; age range, 23–67 years), and healthy participants (males, 53; females, 47; age range, 25–67 years). The patients included were from the National Institute of Diabetes and Endocrinology inpatient and/or the outpatient clinic, Al-Qasr Al-Aini, Cairo, Egypt. The patients and control were from both sexes. Recruitment was based on their medical records, and selection was carried out following the American Diabetes Association diagnostic criteria that specify fasting serum glucose (FSG) ≥ 126 mg/dL or 2 h postprandial serum glucose (PSG) ≥ 200 mg/dL [27]. 

Full medical history, blood pressure (BP), and anthropometric measurements were recorded for each participant, including waist circumference (cm); weight (kg); height (m); and BMI calculation. Patients with the following criteria were excluded from the study: pre-diabetics, type 1 diabetics, and pregnant women. All patients taking vitamin D supplements and all patients with one or more of the following co-morbidities were excluded: end-stage renal disease (ESRD), end-stage liver disease (ESLD), cancer, after organ transplant, early diabetes, or cardiac episode 3 months before trial. A BMI (the weight in kilograms/the height square in meters) of 25 to 29.9 is deemed as “pre-obesity,” and a BMI of >=30 is classified as obesity with classes I-III [28].

### 2.2. Sample Collection

Ten ml venous blood samples were collected from participants in the morning, after 10 h of fasting; 2 mL in an EDTA tube for calculation INR (International Normalized Ratio), 2 mL in a citrate tube for complete a blood picture (CBC), and the remaining volume (6 mL) in 2 plain tubes. Plain tubes were immediately centrifuged, 2 aliquots of serum were frozen at −80 °C for Pro-NT & 25-hydroxy vitamin D3 (25 (OH) D3) analysis using conventional Enzyme-Linked Immuno-Sorbent Assay (ELISA); ELISA sandwich kit for Pro-neurotensin, Cat. No E1318Hu, and Flughafenstrasse. 52a, D-22335 Hamburg, Germany with Cat. -No: RE53041 for 25-OH-vitamin D3. The rest of the serum was immediately analyzed for other analytes. Immediately after the collection of the fasting sample, all participants received 75 g of anhydrous glucose dissolved in 200 mL of water, and 2 mL of blood in a plain tube was collected 2 h after administration. The second sample was immediately centrifuged and was analyzed for post-prandial blood glucose. The fasting blood glucose test, postprandial blood sugar test, fasting blood insulin, kidney function test (urea and creatinine), lipid profile (Cholesterol, TG, Chol-LDL, Chol-HDL), and Hemoglobin A1c were analyzed immediately using an Olympus AU4 (400) instrument. Prothrombin time and INR (International Normalized Ratio) were assessed using KCL Apparatus, and a complete blood picture (CBC) was analyzed using a Phoenix Instrument. The Insulin resistance assessment was carried out with homeostasis model assessment (HOMA-IR) = fasting glucose (nmol/L) multiplied by fasting insulin (microU/L) divided by 22.5 [29].

### 2.3. Ethics Approval

All participants signed the Informed Consent form, and the study protocol was approved by the Ethics Committee of the General Organization of the Hospitals and Educational Institutes (GOTHI) (No: IDE00287). 

### 2.4. Statistical Analysis of Data

The SPSS program (Statistical Package for Social Science) version 27.0 (IBM Crop, 2020 Armonk, NY, USA) was used to analyze the collected data. Data were presented in tables and figures. Quantitative variables were presented as mean ± SD, median (Inter quartile range [IQR]), and range. Frequencies and relative percentages represented qualitative data. The qualitative variables difference was calculated using a Chi-square test. In normally distributed data among both groups, the quantitative variables difference was calculated by independent *t*-test. However, in data that were not normally distributed, the Mann–Whitney (MW) test was used. Moreover, quantitative variables links were calculated using Pearson’s correlation coefficient, and Spearman’s correlation coefficient. Optimal cut-off values of Pro-NT& 25-OH Vit D3 were identified by curve analysis of receiver operating characteristic (ROC) for the expectation of the outcome. A *p*-value < 0.05 was reflected in statistically significant data in the tests.

## 3. Results

### 3.1. Demographic and Clinical Features of the Study Population

The demographic features and biochemical results of the 200 participants included in this study are shown in Table 1. Among the biochemical parameters, T2DM patients had a statistically significant rise in values of HbA1c, FSG, PP serum glucose, fasting insulin, HOMA IR, INR, creatinine, urea, cholesterol, TG, and LDL compared to the control group. T2DM patients also had significantly lower HDL compared to the control group, *p* < 0.001 (Table 1).

There was no significant difference between the two groups in terms of age, sex, BMI, or smoking; however, a predominance of males was noted in both groups. There was a significant difference noted between the two groups’ waist circumferences, with the value being greater in patients with T2DM. Mean systolic and diastolic BP were recorded in T2DM patients as 122.2 ± 14.64 mmHg, and 73.74 ± 10.72 mmHg, respectively, while the measurements of the control group were 112.73 ± 9.97 mmHg and 69.52 ± 9.33 mmHg, respectively. Different percentages of hypertension stages were recorded, with higher percentages in the diabetic group than in the control group.

### 3.2. Comparison in Pro-NT and 25-OH Vitamin D3 between Both Groups

Our results showed that the two groups had a significant difference in pro-NT levels, with mean values of 144.65 ± 48.76 in the T2DM groups and 85.43 ± 30.23 in the control group, *p* < 0.001. Moreover, a significant difference was also noted in the levels of 25-Hydroxy vitamin D3 between both groups, with levels being lower among the T2DM patients, 17.03 ± 8.77, than in the control group, 39.32 ± 13.1, *p* < 0.001 (Table 2).

T2DM has different complications at both micro- and macro-vascular levels. The most frequent complications among our patient population were retinopathy and neurological complications (15% and 12%, respectively) (Table 3).

**Figure 1:**
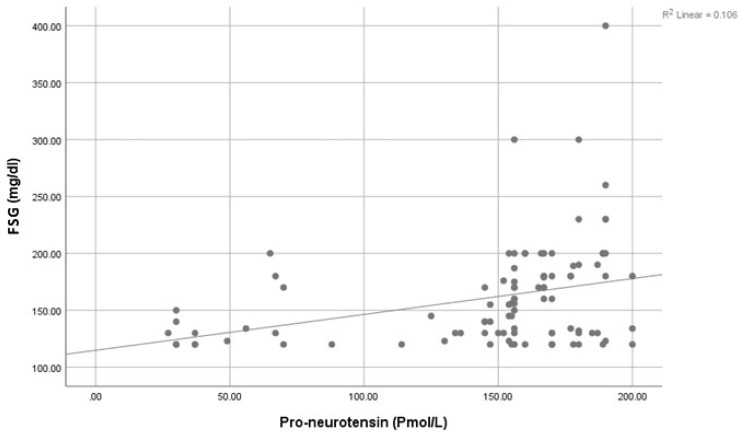
Correlation between Proneurotensin and FSG.

**Figure 2:**
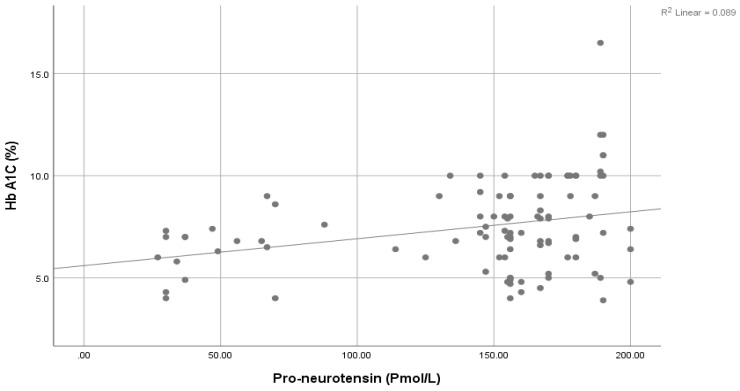
Correlation between Proneurotensin and HbA1c.

**Figure 3:**
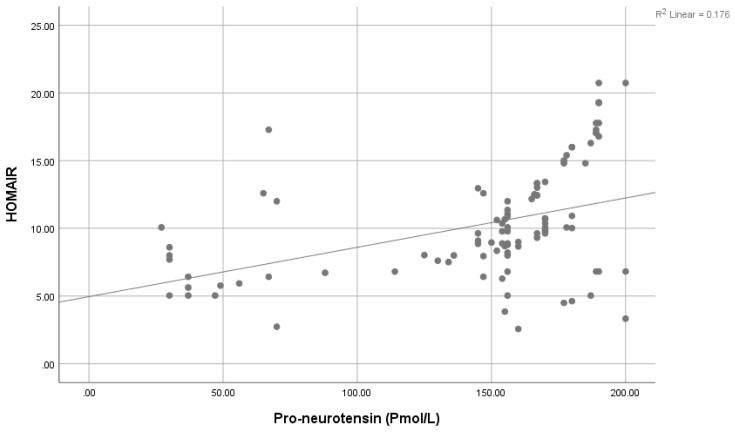
Correlation between Proneurotensin and HOMA-IR.

**Figure 4:**
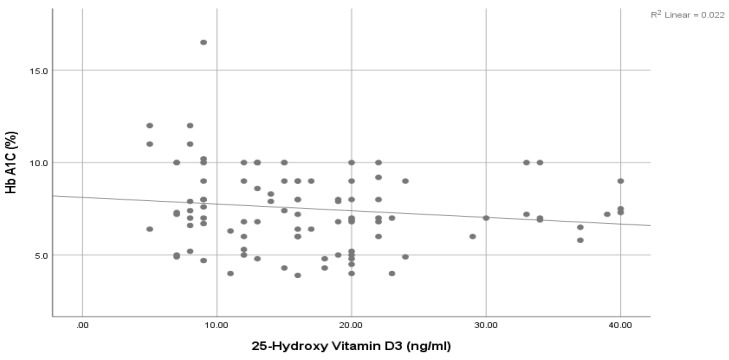
Correlation between 25 (OH) Vit D3 and HbA1c.

**Figure 5:**
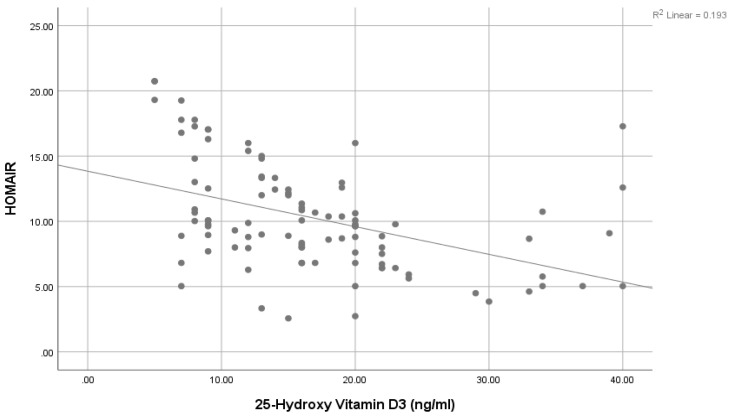
Correlation between 25 (OH) Vit D3 and HOMA-IR.

**Figure 6:**
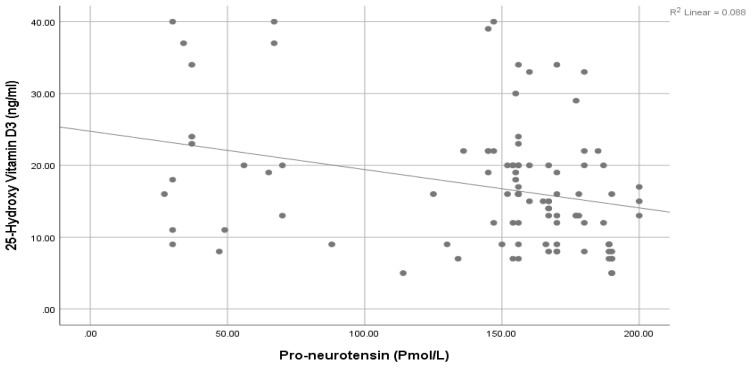
Correlation between Pro-NT and 25 (OH) Vit D3.

A significantly positive association was seen between Pro-NT levels and fasting serum glucose (FSG) (Figure 1), HbA1c (Figure 2), and HOMA-IR (Figure 3). However, a statistically significant negative association was noticed between 25-Hydroxy vitamin D3 and HbA1c (Figure 4) & HOMA-IR (Figure 5). Moreover, a negative association was also seen between 25-Hydroxy vitamin D3 and Proneurotensin (Pro-NT) (Figure 6).

We found that a statistically significant increase in Pro-NT was seen among cases that had retinopathy compared to those that did not. In contrast, cases that had retinopathy and coronary artery complications had a significant decrease in 25 (OH) vitamin D3 compared to those who did not have these complications (Table 4).

In regression analysis of variables which are considered risk factors associated with the complications, we found that fasting serum glucose, HbA1c, and HOMA-IR were positively correlated with the complications as well as Pro-NT. Conversely, 25-Hydroxy vitamin D3 was negatively correlated with diabetic complications (Table 5). 

### 3.3. Determination of the Prediction Value of Pro-NT and 25-OH Vitamin D3 for T2DM

The plasma levels of 25-OH vitamin D3 were significantly lower (*p* < 0.001) in T2DM patients than the control group (Median (IQR) 16 (9–20) and 38 (30.75–44), respectively). The optimal cutoff value of 25-Hydroxy vitamin D3 was <29.5 and the area under the curve (AUC) was 0.94 (95% CI: 0.9–0.97), with 88% sensitivity and 93% specificity (Table 6). The plasma level of Pro-NT was significantly higher (*p* < 0.0001) in patients with T2DM than in the control (Median (IQR) 156 (145–177) and 80 (70–98), respectively). We used Pro-NT >124 Pmol/L as the optimal cutoff value with AUC 0.83 (95% CI: 0.76–0.89) to predict T2DM, with 81% sensitivity and 88% specificity (Table 6).

## 4. Discussion

Based on the IDF report of 2021, in the Middle East and North African Region one in six adults has diabetes, with the number expected to increase by 86% by 2045. Moreover, 30% of diabetic patients remain undiagnosed until their condition is discovered accidentally. Management of diabetes required about 32.6 billion US dollars in 2021 [5]. Early diagnosis of the disease, among other factors, will have a significant impact on disease course and mortality. Thus, better and more reliable markers must be identified to diagnose the disease early in its course.

In our study, cholesterol, triglyceride, and LDL were higher in the diabetic group than in the control group with independent *t* test = 5.47, 12.78 and 6.29, respectively, at *p* < 0.001. This agrees with Khan et al., who also mentioned that HbA1c, which showed a significant rise in T2 diabetic patients, is not only a useful biomarker for long-term glycemic control but is also a good predictor of lipid profile [30]. HbA1c had a statistically significant positive correlation with Pro-NT and a statistically significant negative correlation with 25 (OH) Vit D3.

In tests, the use of vitamin D is continuously correlated with different metabolic conditions beyond bone and mineral metabolism. In our study of 200 participants (100 patients with T2DM and 100 healthy volunteers), a statistically significant decrease in 25-Hydroxy vitamin D3 was seen among diabetic cases compared to the control group with mean values of 17.03 ± 8.77 and 39.32 ± 13.1, respectively, at *p* < 0.001 (Table 2). This supports the results of other studies conducted among different ethnic groups [22,30,31,32,33]. Two studies conducted in 2022 reported that vitamin D supplementations significantly lowered the development of diabetes or at least improved glycemic control among diabetic patients [34,35]. This raises the benefit of 25 (OH) Vit D3 assessment, not only for differentiation between diabetics and healthy populations but also to allow for correction of the course of the disease. This agrees with our results that demonstrate a significantly negative association between 25 (OH) Vit D3 and HOMA IR and HbA1c, which provides a guide for a simple and easy protocol for delaying the onset of T2DM and controlling hyperglycemia by correcting vitamin D deficiency if present. This consecutively decreases insulin resistance and hyperglycemia, both of which are important elements in diabetes pathogenesis and the development of complications. According to our results, a decrease in 25-OH Vit D3 levels in addition to an increased waist circumference (cm) among T2DM cases agrees with previous studies which reported that vitamin D is proficiently stored in the body fat reserves, where it is no longer biologically available, which describes why a substantial number of obese people are severely deficient in vitamin D [26,36].

There have been different opinions on the optimal cutoff points for vitamin D levels in healthy people [37,38,39,40,41]. Our results reveal that at a cutoff value of <29.5, 25 (OH) vitamin D3 shows 88% sensitivity and 93% specificity in predicting T2DM, AUC = 0.94 (95% CI: 0.9–0.97). This cutoff is classified as insufficient 25 (OH) vitamin D3 in many studies [34,35,36], while some other studies report that a Vitamin D level of 40 ng/mL is needed to reduce the risk of many chronic diseases [42,43].

Vitamin D concentration also correlates with diabetic complications, such as myocardial ischemia [44]. In our study, there was a statistically significant decrease in 25 (OH) vitamin D3 among T2DM cases having retinopathy and coronary artery complications, compared to those in which these complications were absent. A lower cutoff value of 25 (OH) vitamin D3, <16.5, with sensitivity and specificity of 67.6% and 62.2 %, respectively, proved for differentiation between patients with and without the complications, AUC = 0.63 (95% CI: 0.52–0.75). This again adds to the benefits of measurement of Vitamin D concentration and early Vitamin D supplementation in the course of the disease.

Our study shows that lower values of 25-OH Vit D3 and higher values of pro-NT, with mean ± SD values of 16.43 ± 8.97 and 17.38 ± 8.70, respectively, showed no significant difference between males and females. However, a correlation between gender and these levels cannot be excluded as shown by Kampmann, U et al. [45]. Moreover, Pang Z et al. also demonstrates a different cutoff of Vitamin D based on gender [46].

The Malmö Diet and Cancer Study 2012 showed that Pro-NT is positively linked to early DM occurrence [12]. This was also supported by our study which showed that Pro-NT levels were significantly higher among the diabetic population compared to the control group, 144.65 ± 48.76 and 85.43 ± 30.23, respectively, at *p* < 0.001. An observational trial by Fawad A et al. indicated that Middle Eastern ethnicity origin has an impact on the relationship between Pro-NT and other indices of glycemic control, which they used as a basis to explain why the Middle Eastern population has a higher potential to suffer from T2DM than North European natives [13].

The significant positive correlation between Pro-NT levels and HbA1c & HOMA IR shown by our study is also demonstrated by Barchetta et al. who showed a positive link between Pro-NT and HOMA-IR in patients with nonalcoholic fatty liver disease [15]. One study demonstrated that follow-up of obese children for eight years shows a significant decrease in insulin secretion to compensate for the decreased insulin sensitivity even though none of them developed T2DM [47]. Additionally, Tönjes et al. showed a free link between Pro-NT and HOMA-IR as an insulin resistance indicator [48]. This strengthens the hypothesis of proneurotensin as an insulin resistance indicator even without the development of diabetes. Intensive management could effectively delay the onset of glycemic imbalance and diabetic complications once insulin resistance has been diagnosed. Additionally, we observed negative correlations between 25-Hydroxy vitamin D3 levels and HOMA-IR values, something which contrasts with the positive correlations found between Pro-NT levels and HOMA-IR values. We therefore deduced that low levels of 25-Hydroxy vitamin D3 and high levels of Pro-NT in T2DM are related to insulin resistance (increased HOMA-IR values).

We found a statistically significant increase in Pro-NT among our patients who had retinopathy, a critical diabetic complication, compared to patients free from this complication. No other trials have reported such comparisons. Other follow-up studies showed a strong link between high pro-NT levels and adverse metabolic profile risk, and cardiovascular events [49,50]. There was a statistically significant increase in mean systolic and diastolic BP among the T2DM patients’ group in comparison with the control group. Elevated arterial BP contributes to an increased incidence of both micro and macro-vascular complications in patients with T2DM [51]. Pro-NT implies various cardio-vascular effects on HR, BP, and heart contractility [52], and the high level of proneurotensin in a diabetic patient may contribute to higher blood pressure. This highlights the need for further investigation into how pro-NT alters endothelial function in diabetic patients.

In our study, the cutoff for Pro-NT as a diagnostic marker to differentiate between T2DM and the control group was >124 pmol/L, a sensitivity of 81.0%, and a specificity of 88.0%, AUC = 0.83. To predict the complications of T2DM, the cutoff value was higher at >158 Pmol/L and AUC = 0.62 (95% CI: 0.50–0.74), with 64.9% sensitivity and 63.5% specificity. Although multiple studies have established a link between proneurotensin and T2DM, the appropriate cutoff for Pro-NT as a T2DM marker is yet to be determined.

This research had some limitations, including the relatively small number of participants, and a bigger sample size is required in the future for optimum results. Additionally, our study was conducted in a cohort case-control design, so a cause-effect link cannot be established. Furthermore, follow-up is required to assess the appropriateness of the cutoff value at the outset of T2DM diagnosis and various stages of complications.

## 5. Conclusions

Pro-NT and 25 (OH) vitamin D3 demonstrate significant validity and accuracy in predicting T2DM (84.5% and 90.5%, respectively). Furthermore, these markers also demonstrated high sensitivity and specificity in predicting T2DM complications. A greater prospective investigation is needed to evaluate the utility of these markers in the early detection of T2DM before the onset of hyperglycemia and complications.

## 6. Study Limitations

Our study is limited by the exclusion of the following criteria from the study: pre-diabetics, type 1 diabetics, and pregnant women. All participants taking vitamin D supplements were excluded, as were patients with one or more of the following co-morbidities: end-stage renal disease (ESRD), end-stage liver disease (ESLD), cancer, after organ transplant, early diabetes, or cardiac episode 3 months before trial. The numbers of the commonly noticed diabetic complications were the actual, real data, and they seem relatively small but reliable. This study is self-funded, so the authors could not afford more funds to study a larger number of patients. Further ongoing large studies may be needed. This research had some limitations, including the relatively small number of participants; a bigger sample size is required in the future for optimum results. Additionally, our study was conducted in a cohort case-control design, so a cause-effect link cannot be established. Furthermore, follow-up is required to assess the appropriateness of the cutoff value at the outset of T2DM diagnosis and at various stages of complications. A study with a depth of focus including multiethnic groups will also be helpful in making an accurate decision.

## Figures and Tables

**Table 1:** Comparison Between the Two Studied Groups Regarding Demographic, Clinical, and Laboratory Data.

Variable		Group I (*n* = 100) (Diabetic)		Group II (*n* = 100) (Control)		T	*p*
Age: (years)	Mean ± SD	48.19 ± 12.24		44.66 ± 11.48		1.98	0.06 NS
	Range	23–67		25–67			
Duration of T2DM: (years)	Mean ± SD	7.62 ± 4.67					
	Range	0.17–20					
BMI: (kg/m^2^)	Mean ± SD	29.85 ± 4.38		28.77 ± 4.61		1.7	0.09 NS
	Range	20–40		20–38			
SBP: (mmHg)	Mean ± SD	122.2 ± 14.64		112.73 ± 9.97		5.35	<0.001 **
	Range	100–160		90–152			
DBP: (mmHg)	Mean ± SD	73.74 ± 10.72		69.52 ± 9.33		2.97	0.003 *
	Range	40–97		50–93			
Waist circumference: (cm)	Mean ± SD	112.88 ± 12.16		104.67 ± 13.09		4.60	<0.001 **
	Range	87–140		80–156			
HbA1c: (%)	Mean ± SD	7.46 ± 2.13		3.33 ± 1.22		16.79	<0.001 **
	Range	3.9–16.5		1.2–5			
FSG: (mg/dL)	Mean ± SD	160.95 ± 45.22		85.23 ± 8.84		14.13	<0.001 **
	Range	120–400		67–102			
Post prandial serum glucose: (mg/dL)	Mean ± SD	251.4 ± 72.11		119.43 ± 14.9		17.92	<0.001 **
	Range	140–600		90–177			
HOMA IR:	Mean ± SD	10.31 ± 3.15		4.44 ± 1.42		13.4	<0.001 **
	Range	2.57–20.74		1.91–6			
INR:	Mean ± SD	1.23 ± 0.18		1.07 ± 0.10		7.6	<0.001 **
	Range	1–1.73		1–1.4			
Creatinine: (mg/dL)	Mean ± SD	1.01 ± 0.19		0.95 ± 0.16		2.21	0.03 *
	Range	0.6–1.5		0.7–1.2			
Urea: (mg/dL)	Mean ± SD	34.79 ± 10.67		29.8 ± 8.34		3.68	<0.001 **
	Range	20–67		13–55			
Cholesterol: (mg/dL)	Mean ± SD	172.78 ± 41.01		148.12 ± 18.75		5.47	<0.001 **
	Range	87–280		115–203			
Triglyceride: (mg/dL)	Mean ± SD	189.77 ± 24.76		146.72 ± 22.84		12.78	<0.001 **
	Range	120–270		121–220			
Chol-HDL: (mg/dL)	Mean ± SD	32.88 ± 9.93		43.83 ± 8.70		8.29	<0.001 **
	Range	20–60		30–88			
Chol-LDL: (mg/dL)	Mean ± SD	126.75 ± 27.83		107.94 ± 10.99		6.29	<0.001 **
	Range	99–202		89–134			
Variable		No	%	No	%	χ^2^	*p*
Sex:	Male	63	63%	53	53%	2.05	0.15
	Female	37	37%	47	47%		NS
Smoking:	No	84	84%	79	79%	0.83	0.36
	Yes	16	16%	21	21%		NS
Hypertension stage:	Normal	50	50%	84	84%	27.12	<0.001 **
	Pre-hypertensive	30	30%	12	12%		
	Stage 1	19	19%	4	4%		
	Stage 2	1	1%	0	0%		

SD: Standard deviation, Test: Independent *t*-test, χ^2^: Chi-square test, NS: Non-significant (*p* > 0.05), *: Significant (*p* < 0.05), **: highly significant (*p* < 0.001), T2DM: type 2 diabetes mellitus, BMI: body mass index, LDL: low-density lipoprotein, FSG: fasting serum glucose, INR: international normalized ratio, HDL: high-density lipoprotein, HOMA IR: insulin resistance assessment using homeostasis model assessment, HbA1c: Glycosylated Hemoglobin, Type A1C.

**Table 2:** Pro-neurotensin Levels Versus 25-Hydroxy Vitamin D3 Levels among The Studied Groups.

Variable		Group I (Diabetic) (*n* = 100)	Group II (Control) (*n* = 100)	Mann–Whitney Test	*p*
Pro-neurotensin: (pmol/L)	Mean ± SD	144.65 ± 48.76	85.43 ± 30.23	7.97	<0.001 **
	Range	27–200	34–180		
	Median (IQR)	156 (145–177)	80(70–98)		
25-Hydroxy vitamin D3: (ng/mL)	Mean ± SD	17.03 ± 8.77	39.32 ± 13.1	10.67	<0.001 **
	Range	5–40	12–98		
	Median (IQR)	16 (9–20)	38 (30.75–44)		

SD: Standard deviation, IQR: Inter quartile range, **: highly significant (*p* < 0.001).

**Table 3:** Percentage of Complications in the Diabetic Group.

Complication	Group I (Diabetic) (*n* = 100)	
	Number	%
Retinopathy:	15	15
Neurological complications:	12	12
Coronary artery diseases:	8	8

**Table 4:** Correlation between Pro-NT and 25 (OH) Vit D3 with Diabetic Complications among the Studied Cases Group.

Complication		Pro-Neurotensin						25 (OH) Vitamin D3					
		N	Mean	SD	Median	MW	*p*	N	Mean	SD	Median	MW	*p*
Retinopathy	No	85	140.01	49.58	156	3.01	0.003 *	85	18.09	8.9	16	3.12	0.002 *
	Yes	15	170.93	34.55	189			15	11	4.8	9		
Neurological complications	No	88	145.45	48.4	156	0.14	0.89 NS	88	17.01	8.64	17	0.19	0.85 NS
	Yes	12	138.75	53.18	145			12	17.17	10.09	15		
Coronary artery diseases	No	92	143.15	48.82	156	1.69	0.09 NS	92	17.61	8.84	16	2.48	0.01 *
	Yes	8	161.88	47.64	178			8	10.38	4.17	10		

N: number, SD: Standard deviation, MW: Mann–Whitney test, NS: Non-significant (*p* > 0.05), *: Significant (*p* < 0.05).

**Table 5:** Linear regression analysis for factors associated with complication occurrence among the studied cases.

	Standardized Coefficients	*p*	95.0% Confidence Interval for B	
	Beta			
Sex: (m/f)	−0.092	0.364	−0.264	0.098
Age: (year)	−0.124	0.240	−0.012	0.003
BMI: (kg/m^2^)	−0.041	0.699	−0.025	0.017
Disease duration: (year)	−0.081	0.436	−0.027	0.012
Waist circumference: (cm)	0.011	0.918	−0.007	0.008
SBP: (mmHg)	0.023	0.850	−0.006	0.008
DBP: (mmHg)	0.016	0.892	−0.009	0.010
Fasting serum glucose: (mg/dL)	**0.614**	0.002 *	0.601	1.006
Post prandial serum glucose: (mg/dL)	−0.033	0.937	−0.053	0.048
Fasting insulin: (mIU/L)	0.529	0.257	−0.004	0.014
HbA1c: (%)	0.640	0.041 *	0.604	1.040
HOMA-IR:	0.228	0.037 *	0.033	1.750
INR:	0.190	0.750	−0.105	0.145
Creatinine: (mg/dL)	0.049	0.671	−0.002	0.003
Urea: (mg/dL)	0.079	0.448	−0.005	0.012
Cholesterol: (mg/dL)	0.055	0.629	−0.002	0.003
Triglyceride: (mg/dL)	0.150	0.229	−0.002	0.007
Chol-HDL: (mg/dL)	0.252	0.015	0.002	0.020
Chol-LDL: (mg/dL)	−0.056	0.656	−0.005	0.003
Pro-neurotensin: (Pmol/L)	0.351	0.002 *	0.309	1.311
25-Hydroxy vitamin D3: (ng/mL)	−0.369	0.006 *	−0.013	−0.010

*: Significant (*p* < 0.05); BMI: body mass index; SBP: systolic blood pressure; DBP: diastolic blood pressure; HDL: high-density lipoprotein; LDL: low-density lipoprotein; HOMA-IR: insulin resistance assessment using homeostasis model assessment; INR: international normalized ratio; HbA1c: Glycosylated Hemoglobin: Type A1C.

**Table 6:** Receiver Operating characteristic (ROC) curve of Pro-NT and 25 (OH) Vitamin D3 levels in the prediction of T2DM among the studied groups.

Variable	Cut Off	AUC (95% CI)	Sensitivity	Specificity	PPV	NPV	Accuracy	*p*
Pro-neurotensin: (Pmol/L)	>124	0.83 (0.76–0.89)	81%	88%	87.1%	82.2%	84.5%	<0.001 **
25-Hydroxy vitamin D3: (ng/mL)	<29.5	0.94 (0.9–0.97)	88%	93%	92.6%	88.6%	90.50%	<0.001 **

AUC: Area under the curve, CI: Confidence interval, PPV: Positive predicted Value, NPV: Negative predicted value, **: highly significant (*p* < 0.001).

## Data Availability

Data is available upon request from the first and corresponding author.
